# Supplementary material for: Selections that isolate recombinant mitochondrial genomes in animals
Source: eLife. 2015 Aug 3;4:e07247. doi: 10.7554/eLife.07247 (PMC4584245; doi:10.7554/eLife.07247)
Supplement: Supplementary file 1. — HR-based repair was also able to rescue in somatic tissues like eye. High-level eye-antennal disc expression of both mito-BglII and mito-XhoI using ey-Gal4 at 29°C resulted in pupal lethality (headless pupa) in wild-type flies or in flies carrying mt genomes resistant to only one of the two enzymes. A few (about 1%) escapers eclosed and the majority of eclosed flies had no eye or small eye phenotype (rows 1 and 2). Having both genomes increased the eclosion rate to 10% (row 3) and most of the eclosed flies had normal-sized eyes (not shown). DOI: http://dx.doi.org/10.7554/eLife.07247.010 [file elife07247s001.docx]

**Supplementary file 1**: HR-based repair was also able to rescue in somatic tissues like eye. High-level eye-antennal disc expression of both mito-BglII and mito-XhoI using ey-Gal4 at 29°C resulted in pupal lethality (headless pupa) in wild-type flies or in flies carrying mt genomes resistant to only one of the two enzymes. A few (about 1%) escapers eclosed and the majority of eclosed flies had no eye or small eye phenotype (rows 1 and 2). Having both genomes increased the eclosion rate to 10% (row 3) and most of the eclosed flies had normal sized eyes (not shown).

| Mitochondrial genotype | Total # of pupa | Total # of flies eclosed | % of flies eclosed |
| --- | --- | --- | --- |
| mt:CoI^R301Q^ | 584 | 7 | 1.20 |
| mt:ND2^del1^ | 420 | 4 | 0.95 |
| mt:ND2^del1^/ mt:CoI^R301Q^ | 418 | 42 | 10.05 |
