# Supplementary material for: Selections that isolate recombinant mitochondrial genomes in animals
Source: eLife. 2015 Aug 3;4:e07247. doi: 10.7554/eLife.07247 (PMC4584245; doi:10.7554/eLife.07247)
Supplement: Supplementary file 2. — A list of primers used in this study. DOI: http://dx.doi.org/10.7554/eLife.07247.011 [file elife07247s002.docx]

**Supplementary file 2**: A list of primers used in this study

| **Primers** | **Sequences** | **Function** |
| --- | --- | --- |
| mt186F | ttaagctactgggttcatacccc | Sequencing fragment I |
| mt714F | attattggagctattggaggact | Sequencing fragment I |
| mt1579F | cgagctgaattaggacatcc | Sequencing fragment I |
| mt2126F | ttgacccagcgggaggaggagat | Sequencing fragment I |
| mt2884F | tgagaaagtttagtatcacaacga | Sequencing fragment I |
| mt3567F | cttgaacagtacctgctttagg | Sequencing fragment I |
| mt4807R | agctcctgttaatggtcatggac | Sequencing fragment I |
| mt4583F | tagctgcaggtaaccaagaag | Sequencing fragment I |
| mt5331F | ttatagcaacaggattccacgg | Sequencing fragment I |
| mt6115F | tctttaattgaagccaaaaagagg | Sequencing fragment I |
| mt6905F | ccgcaatttaaatcaccgg | Sequencing fragment II |
| mt7512F | tagctgcaggtaaccaagaag | Sequencing fragment II |
| mt7963F | acttattcaatcaaaaagaaaagttataac | Sequencing fragment II |
| mt8701F | tatgagcaacagatgaataagc | Sequencing fragment II |
| mt9382F | cacaacctaaaaaataagaaatttctgatc | Sequencing fragment II |
| mt10358F | ctttatctttaaataaattatataattttcccac | Sequencing fragment II |
| mt11003F | ttgctgttgataatgccac | Sequencing fragment II |
| mt11517F | agctcgaccagttgaagaacc | Sequencing fragment II |
| mt12043F | cagcaaaatcaaaaggattccg | Sequencing fragment II |
| mt12822F | aaccaacctggcttacacc | Sequencing fragment II |
| mt13296F | cgtccaaccattcattccagcc | Sequencing fragment II |
| mt14797R | gtgccagcagtcgcggttatac | Sequencing fragment II |
| **PCR primers** |  |  |
| mt186F | ttaagctactgggttcatacccc | F primer for fragement I |
| mt7524R | ttacctgcagctatagctgctc | R primer for fragement I |
| mt6905F | ccgcaatttaaatcaccgg | F primer for fragement II |
| mt14797R | gtgccagcagtcgcggttatac | R primer for fragement II |
| **Southern primers** | | |
| mt1579F | cgagctgaattaggacatcc |  |
| mt2365R | ctacatctattccaacggtaaatata |  |
